# Milk-Compositional Study of Metabolites and Pathogens in the Milk of Bovine Animals Affected with Subclinical Mastitis

**DOI:** 10.3390/molecules27238631

**Published:** 2022-12-06

**Authors:** Aarif Ali, Manzoor Ur Rahman Mir, Showkat Ahmad Ganie, Saima Mushtaq, Sarah I. Bukhari, Sultan Alshehri, Shahzada Mudasir Rashid, Tahir Maqbool Mir, Muneeb U. Rehman

**Affiliations:** 1 Department of Clinical Biochemistry, School of Biological Sciences, University of Kashmir, Srinagar 190006, Jammu and Kashmir, India; 2 Division of Veterinary Biochemistry, Faculty of Veterinary Sciences & Animal Husbandry, Sher-e-Kashmir University of Agricultural Sciences and Technology, Kashmir (SKUAST-K), Shuhama Campus (Alusteng), Ganderbal 190006, Jammu and Kashmir, India; 3 Veterinary Microbiology Department, Indian Veterinary Research Institute (IVRI), Bareilly 243122, Uttar Pradesh, India; 4 Department of Pharmaceutics, College of Pharmacy, King Saud University, Riyadh 11451, Saudi Arabia; 5 National Centre for Natural Products Research, University of Mississippi, Oxford, MS 38677, USA; 6 Department of Clinical Pharmacy, College of Pharmacy, King Saud University, Riyadh 11451, Saudi Arabia

**Keywords:** milk, subclinical mastitis, metabolome, microbiome, antibiotic sensitivity test

## Abstract

Bovine milk is an important food component in the human diet due to its nutrient-rich metabolites. However, bovine subclinical mastitis alters the composition and quality of milk. In present study, California mastitis testing, somatic cell count, pH, and electrical conductivity were used as confirmatory tests to detect subclinical mastitis. The primary goal was to study metabolome and identify major pathogens in cows with subclinical mastitis. In this study, 29 metabolites were detected in milk using gas chromatography–mass spectrometry. Volatile acidic compounds, such as hexanoic acid, hexadecanoic acid, lauric acid, octanoic acid, *n*-decanoic acid, tricosanoic acid, tetradecanoic acid, and hypogeic acid were found in milk samples, and these impart good flavor to the milk. Metaboanalyst tool was used for metabolic pathway analysis and principal component estimation. In this study, EC and pH values in milk were significantly increased (*p* < 0.0001), whereas fat (*p* < 0.04) and protein (*p* < 0.0002) significantly decreased in animals with subclinical mastitis in comparison to healthy animals. *Staphylococcus aureus* was the predominant pathogen found (*n* = 54), followed by *Escherichia coli* (*n* = 30). Furthermore, antibiotic sensitivity revealed that *Staphylococcus aureus* was more sensitive to gentamicin (79.6%), whereas *Escherichia coli* showed more sensitivity to doxycycline hydrochloride (80%).

## 1. Introduction

Subclinical mastitis (SCM) generally refers to a multiple etiology mammary gland inflammation with bacteria being the most prominent etiological agent. About 140 species of bacteria have been isolated from infected milk samples with staphylococci, streptococci, and enterobacteria being the predominant agents responsible for intramammary infections (IMIs) [1,2]. SCM is often difficult to differentiate from clinical mastitis (CM), as the former lacks visual disease symptoms like inflammation, pain, fever, clots in milk, anorexia, and reduced milk yield and quality [3,4]. SCM mostly alters the milk composition and makes it unfit for human consumption. Bovine milk is a highly enriched nutritious entity primarily composed of water, sugars, proteins, lipids, vitamins, and minerals. Besides these, a small number of metabolite molecules are also found in bovine milk [5]. Bovine milk contains a large quantity of lipid molecules and approximately 400 different types of fatty acids have been found, mostly in trace amounts [5]. Lipids in milk are very complex chemically and often exist in a unique emulsion form [5]. As compared to other mammals, ruminant milk contains unique fatty acids, e.g., butyric acid and conjugated linoleic acid. In dairy animals, milk is a promising functional food with health promoting and disease preventing features. In neonates, milk provides protein for nutritional purposes, and to understand the variations that occur in this vital food component during host response to mastitis is important.

In dairy animals, metabolomic approaches have mostly been used to determine dynamic metabolic responses with various pathways linking a series of chemical reactions occurring in a cell. Metabolomic pathways can provide key insights about biological modules related to SCM or IMIs. Recent developments in the metabolomic field has allowed unprecedented growth in understanding metabolite changes in farm animals in a diseased state. In bovine animals, the metabolomics field has added valuable insights on food safety, animal health, and production. Untargeted metabolomic approaches have been important in identifying new metabolite molecules that were previously not recognized, and if validated, these analytes could serve as screening markers for bovine mastitis. The identification of metabolite differences by metabolomic studies has allowed the distinguishing healthy cows from animals with subclinical mastitis. Thus, metabolites could serve as important markers of subclinical mastitis/IMIs in dairy cows.

In dairy animals, the study of the microbiome is gaining much attention, particularly during mastitis infection [6]. Bhatt et al. [7] reported that 99% of environmental microbes cannot be easily cultivated. Within the vicinity of mammary gland, the presence of natural microbial community is known [6]. In mastitis-infected quarters, a wide diversity of microbial genera has been reported [8,9] along with higher bacterial load as compared to uninfected quarters [10]. Therefore, it is very important to study the microbiome and to identify major pathogens associated with SCM. In SCM, antimicrobial drugs are most commonly used for treatment purposes. However, improper antibiotic usage has progressively developed antibiotic-resistant bacteria. Infected milk with mastitis could be a transmission medium for zoonotic diseases, such as tuberculosis, brucellosis, Q-fever, scarlet fever, diphtheria, and thus pose threats to human beings [11,12,13,14]. Hence, it is very important to determine drug sensitivity in the milk samples of dairy cows affected with SCM in order to reduce the menace of drug resistance and to minimize antimicrobial residues presence in milk.

The present study was carried with the aim of detecting metabolites, studying milk biochemistry, and identifying pathogens in infected milk samples along with evaluation of their antibiogram profiles.

## 2. Results and Discussion 

### 2.1. GC-MS Analysis

**Figure 1:**
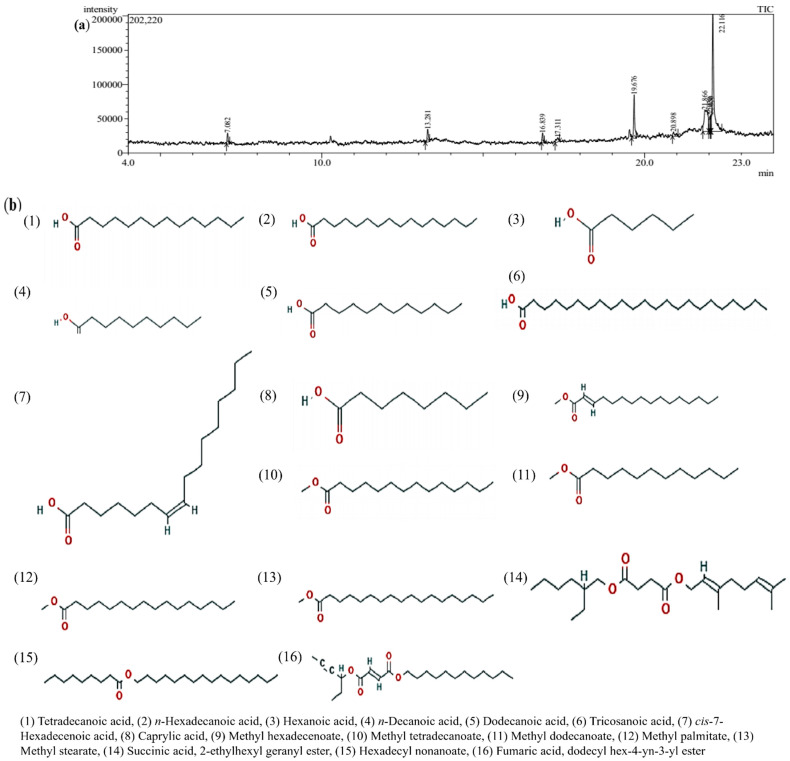

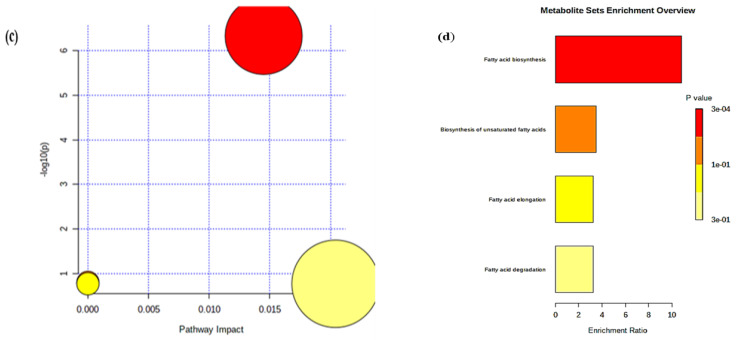
(**a**) Chromatogram obtained from GC-MS of bovine milk; (**b**) structure of compounds found in bovine milk; (**c**) pathway impact of differential milk metabolites of Holstein Friesian cows with MetaboAnalyst; (**d**) summary plot for over-representation analysis (ORA).

A total of 29 metabolites in bovine milk were only detected by a full scan mode of GC-MS analysis that only provides the qualitative picture of composition. In milk samples, the presence of volatile compounds like hexanoic acid (caproic acid), octanoic acid (caprylic acid), hexadecanoic acid (palmitic acid), lauric acid (dodecanoic acid), n-decanoic acid (capric acid), tricosanoic acid, tetradecanoic acid (myristic acid), and hypogeic acid, etc., imparts good flavor to the milk. Table 1 shows the major compounds with their names, retention time, peak, and area %. Figure 1a,b represents the chromatogram obtained and the structure of metabolites found in bovine milk.

A large amount of data were generated by the metabolomics approach through advanced biological and statistics methods. In the present study, fatty acids, such as hexanoic acid, tetradecanoic acid, *n*-hexadecanoic acid, dodecanoic acid, *n*-decanoic acid, tricosanoic acid, cis-7-hexadecenoic acid, and caprylic acid, were found in milk samples, and a qualitative picture was provided using the GC-MS approach. Boudonck et al. [15], who found metabolites, such as caprate (10:0), laurate (12:0), myristate (14:0), palmitate (16:0), stearate (18:0), 5-dodecenoate (12:1), and oleate (18:1), in milk samples, reported similar findings. In milk infected with mastitis, an increase in fatty acids was reported [16]. This is primarily because of the increased lipolysis that raises free fatty acids levels in milk [17]. Elevated levels of fatty acids are expected during the progression of infection, as they serve as a precursor for eicosanoids that have a role in the inflammatory response and ultimately could be beneficial to host immunity [16]. Palmitate, oleate, myristate, and stearate were the main fatty acids found in the milk of dairy cows [18]. In milk samples, medium- to high-level fatty acids were reported by Coppa et al. [19]. Similarly, short chain (C4), very long (23:0), and highly unsaturated fatty acids (22:5n-3) were also found in the milk of dairy cows [20]. In milk, the high level of fatty acids is due to hydrolysis that is mainly caused by bacterial lipases [21]. During intramammary infection, the composition of milk changes, which also includes the milk fat content. In the host, fatty acids can either benefit or cause harm due to their regulatory inflammatory reaction mechanisms [22]. The Toll-like receptor 4 (TLR4) is activated by saturated fatty acids, such as Oleic acid, Lauric acid, and Palmitic acid, which subsequently activate the host’s innate defense system against the invading pathogens [22].

### 2.2. Metabolomic Data Analysis

#### 2.2.1. MetaboAnalyst

MetaboAnalyst 5.0 (Wishart Research Group at University of Alberta, Canada) web server was used to reveal the metabolite differences between the healthy and SCM animals. In the pathway analysis module, a list of compounds (one per row) is entered. The major pathways involved were fatty acid biosynthesis, unsaturated fatty acid biosynthesis, and the degradation of fatty acids.

#### 2.2.2. Compound Mapping

This is an important step as the labelled compounds are compared subsequently with metabolite library set contained compounds. In MSEA, an inbuilt tool is present to convert between common names of compounds, identifiers, synonyms used in PubChem, HMDB, METLIN, and KEGG. The conversion mapping is the first phase to standardize compound labels and is predominantly used during the comparison with the metabolite compound library set (Table 2).

#### 2.2.3. Pathway Analysis

A metabolite library set must be chosen prior to enrichment analysis. In this study, we selected relevant *Bos taurus* library (bta) and found that differential metabolites were involved in the biosynthesis, elongation, and degradation of fatty acids in addition to the synthesis of unsaturated fatty acids. In healthy and SCM dairy animals, several enriched pathways (*p* < 0.05) were detected by using these detected milk metabolites. The metabolic pathways are represented as circles according to their scores from enrichment (vertical axis) and topology analysis (pathway impact, horizontal axis). The pathway impact score is represented by circle size and correlates with the centrality of metabolites involved. The color of each metabolic pathway is associated with the obtained P value from enrichment analysis and its size represents the fold enrichment score, i.e., −ln(P). Figure 1c represents the impact of the pathway.

#### 2.2.4. Metabolite Set Enrichment Analysis

Metabolite sets were significantly enriched in fatty acid biosynthesis pathway with five metabolites (hexadecanoic acid, tetradecanoic acid, dodecanoic acid, decanoic acid, and octanoic acid) and one metabolite (hexadecanoic acid) involved in the elongation/degradation of fatty acid (Table 3). The unsaturated fatty acid pathway involved one metabolite (hexadecanoic acid). The most significant pathway using *Bos taurus* as a library was the fatty acid biosynthetic pathway that involved maximum metabolites. The enrichment ratio for fatty acid synthesis was 10.78, and 3.25 for elongation, 3.257 for degradation, and 3.521 for unsaturated fatty acid biosynthesis. The metabolite sets enrichment overview of top 25 metabolites with different abundances is shown in Figure 1d. The detailed results of the pathway analysis are shown in Table 3, where Total is the total number of compounds in the pathway; Hits is the actual matched number from the user uploaded data; Raw p is the original p value calculated from the enrichment analysis; Holm p is the p value adjusted through the Holm–Bonferroni method; FDR p is the p value adjusted using the false discovery rate; and Impact is the pathway impact value calculated through pathway topology analysis.

**Figure 2:**
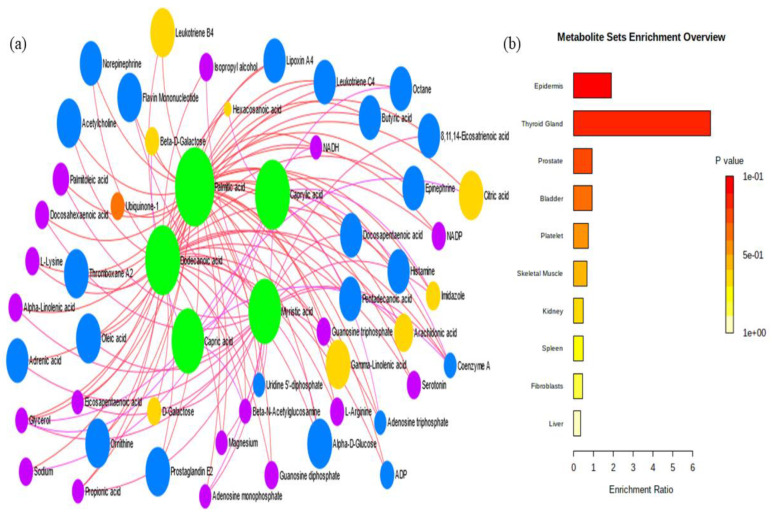
(**a**) Metabolite–metabolite interaction network. The colored circles depict the relationship of metabolites with other metabolite sets. (**b**) Metabolite set enrichment analysis (MSEA) for identified metabolites.

The metabolite–metabolite interactions of five metabolites (hexadecanoic acid, tetradecanoic acid, dodecanoic acid, decanoic acid, and octanoic acid) network showed their potential relationships with other sets of metabolites (Figure 2a).

Metabolite sets were significantly enriched (*p* < 0.05) in epidermis with five metabolites (palmitic acid, caprylic acid, capric acid, dodecanoic acid, and myristic acid) and three metabolites in prostrate (palmitic acid, dodecanoic acid, and myristic acid) as the most significant set (Table 4). Only individual metabolites were found in other organs, such as the thyroid gland (dodecanoic acid), bladder (palmitic acid), platelet (palmitic acid), skeletal muscle (palmitic acid), kidney (palmitic acid), spleen (myristic acid), fibroblasts (palmitic acid), and liver (dodecanoic acid), respectively (Table 4). The metabolite enrichment ratio was highest in thyroid gland (6.897) followed by epidermis (1.923). The MSEA ratio for different organs is as follows: prostrate (0.932), bladder (0.952), platelet (0.769), skeletal muscle (0.676), kidney (0.505), spleen (0.488), fibroblasts (0.455), and liver (0.355) (Figure 2b).

The metabolites identified in milk samples from our study were mainly located in the epidermis and prostrate. Wang and Kadarmideen [23] have also reported metabolites in plasma that were primarily located in the prostrate and mitochondria. As ubiquitous cell organelles, the prostrate, peroxisome, and mitochondria interact with one another, and the latter two play an essential role in metabolic pathways, particularly fatty acid metabolisms, which might be associated with feed utilization [24].

### 2.3. Milk Chemistry

The samples of milk from healthy and SCM animals were analyzed for detailed compositional changes.

#### 2.3.1. Electrical Conductivity (EC) and pH

A significant increase (*p* < 0.0001) in EC and pH values was observed in milk samples of subclinical mastitis animals in comparison to healthy animals.

#### 2.3.2. Milk Fat and Protein

The content of fat in healthy and infected milk was 3.95% and 3.35%, respectively. A significant decrease (*p* < 0.04) was observed in the milk fat percentage of SCM animals as compared to healthy animals. The mean protein percentage in normal milk was 3.51% and that of SCM milk was 3.05%. On average, the protein percentage in milk of subclinical mastitis animals decreased significantly (*p* < 0.0002) as compared to healthy animals.

#### 2.3.3. Lactose and SNF

The mean concentration of lactose was 4.22 and 3.90 in the milk of healthy and subclinical mastitis animals. The mean percentage of solid not fat (SNF) was 7.53% in normal milk and 7.45% in mastitis milk. A non-significant decrease was observed in lactose and SNF in mastitis-infected milk samples in comparison to healthy animals.

#### 2.3.4. Density

The mean concentration of density in milk of healthy and mastitis dairy animals was 25.83 and 24.85, respectively. A significant decrease (*p* < 0.04) was observed in the milk density of mastitis-infected animals as compared to healthy animals.

The mean values, fold changes with *p* values, direction changes in milk composition parameters in SCM-infected animals and healthy animals are shown in Table 5.

In the present study, a significant increase in electrical conductivity and pH in subclinical mastitis milk was observed. Ali et al. [4] reported similar findings. During mastitis, the permeability of the mammary gland tissues increases, which causes ions (sodium, potassium, and calcium) to move from the blood into the milk, thus raising EC and pH. The concentration of these ions (anions and cations) determines the electrical conductivity in milk. When a mammary infection occurs, the integrity of tight junctions is lost, and because of the tissue damage and neutrophil diapedesis caused by mastitis pathogens, the epithelium becomes leaky, and consequently, milk and blood components pass through. The increased EC in the SCM group could be related to ion alterations, specifically an increase in milk sodium and chloride concentrations, due to inflammatory alterations in the mammary tissue. Similarly, the concentration of sodium and bicarbonate (alkaline blood components) is increased, thus raising the milk pH.

In this study, a decrease in the levels of fat percent, lactose, proteins, solid non-fat (SNF), and density in SCM milk was observed. These results agree with the findings of Schukken et al. [25] and Yarabbi et al. [26]. This decrease in various milk components is due to the altered synthesis and secretory activity of mammary epithelial cells caused by the microbial invasion of the udder, which causes inflammatory changes in the udder parenchyma

### 2.4. Principal Component Analysis

**Figure 3:**
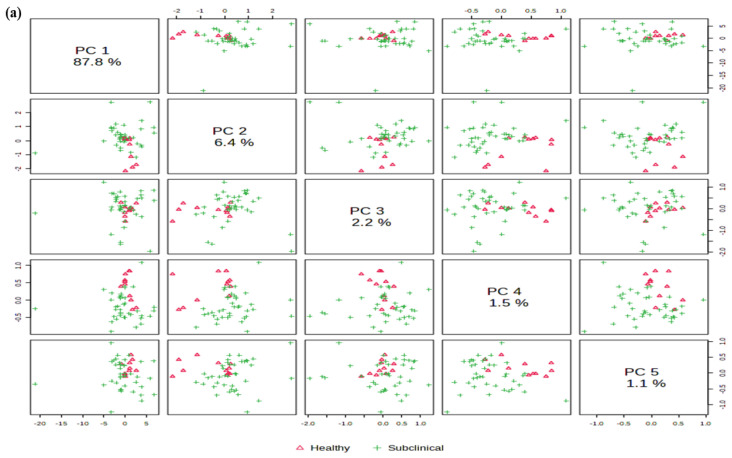

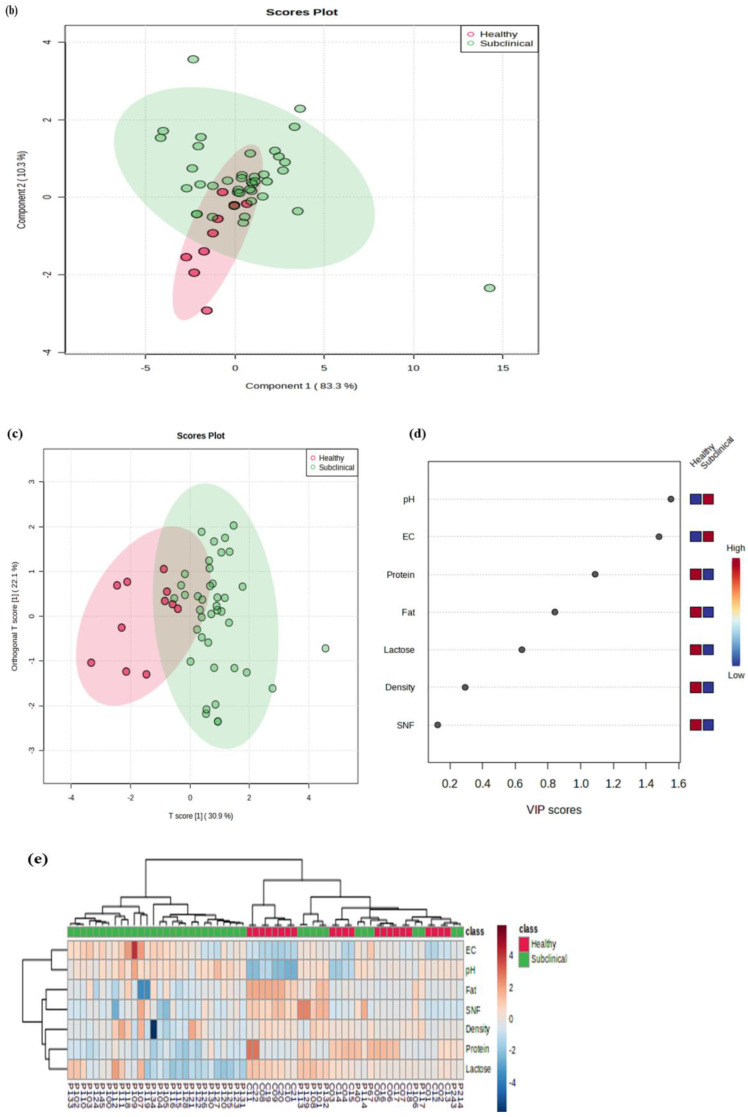
(**a**) PCA score plots of milk metabolites; (**b**) sparse partial least squares discriminant analysis (sPLS-DA) of the metabolites detected in the milk samples of healthy and subclinical mastitis animals. The data have been normalized and represent the mean values of overall samples (0 = Healthy and 1 = Subclinical); (**c**) Orthogonal partial least-squares discriminant analysis (oPLS-DA) score plot of a comparison between healthy and subclinical mastitis groups; (**d**) VIP plot of milk metabolites; (**e**) Hierarchical clustering heat map analysis of milk chemistry metabolite variations between control and subclinical mastitis dairy animals. Data have been normalized with unit variance scaling and the average values of the samples are shown. Comparisons were generated via an average linkage method based on Euclidean distance. Shades from blue to red represent increasing metabolite levels.

The principal component analysis identified five principal components (PCs), PC1, PC2, PC3, PC4, and PC5, that account 87.8%, 6.4%, 2.2%, 1.5%, and 1.1%, respectively, as represented in Figure 3a. The score plot analysis revealed PC1 accounted for maximum variability (87.8%) followed by PC2 (6.4%). To evaluate the performance classification of seven metabolite sets, sPLS-DA revealed two principal components, PC1 and PC2, that accounted for 93.6% variability among the groups, as depicted in Figure 3b.

The classification performance of the PLSDA model was determined based on the function using five-fold cross validation repeated ten times, and the overall and balanced error rates per class were below 0.2 within components. To compare differences of milk chemistry metabolites (fat%, protein%, lactose, SNF, specific density, EC, and pH) between healthy and mastitis-infected animals, we analyzed the 0 (pure milk) and 1 (infected milk) data based on oPLS-DA, the results of which showed that the groups were clustered separately, as shown in Figure 3c. oPLS-DA indicates the similarity and separation of two groups. This evaluation model depicted a high fitting accuracy (*p* < 0.001), using a 1000 times permutation test, and the results revealed 30.9% variation among two groups and thus separated pure milk from mastitis-infected milk (x-axis). Moreover, within the groups, the study analysis demonstrated a difference of 22.1%, thereby showing a rich metabolite variation and a good separation between pure milk and subclinical mastitis milk.

The VIP plots of milk metabolites, as shown in Figure 3d, describe pH, EC, and protein, which are the most important discriminating factors in distinguishing SCM animals from healthy animals. Typically, VIP values > 1 are significant and VIP values > 2 are highly significant.

A heat map was generated within the various spatial milk samples to show the accumulated levels of the top seven contributors, as shown in Figure 3e. Based on heat map graphics, it could be interpreted that many metabolite molecules among the groups depicted a decreasing pattern from the outer to the inner layers. In general, the two groups were found to show similar trends in the spatial distribution of metabolites, although distinct patterns were observed in each group.

**Figure 4:**
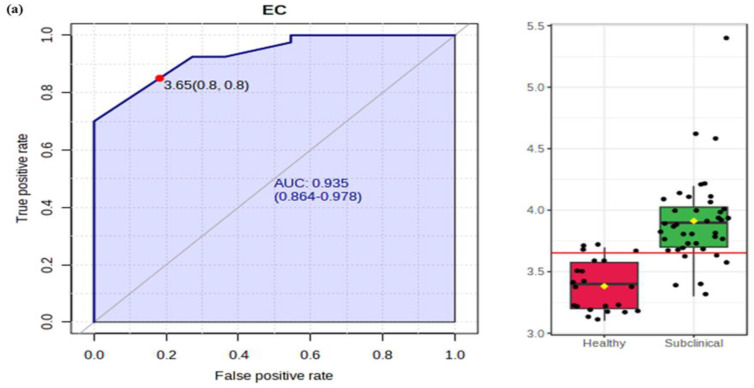

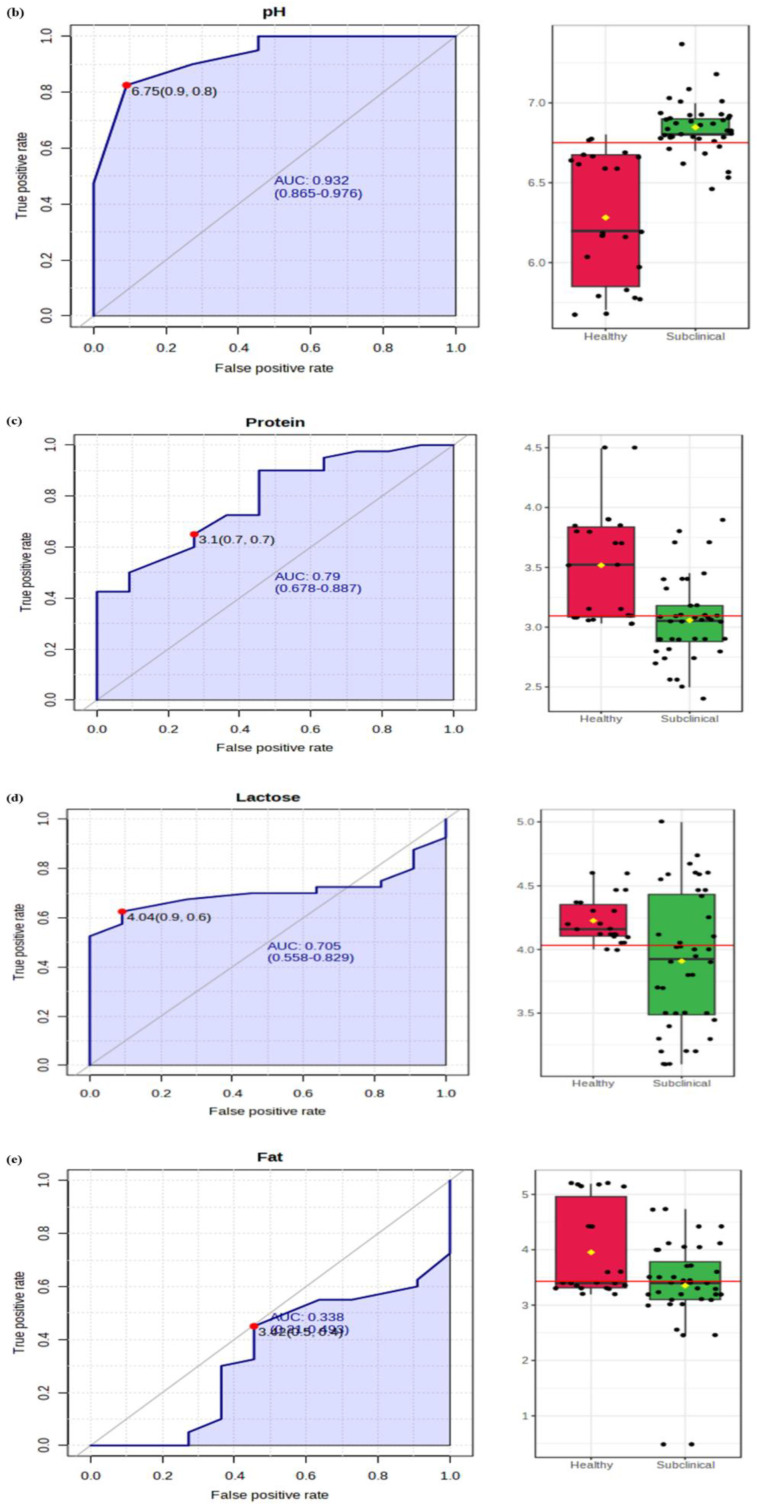

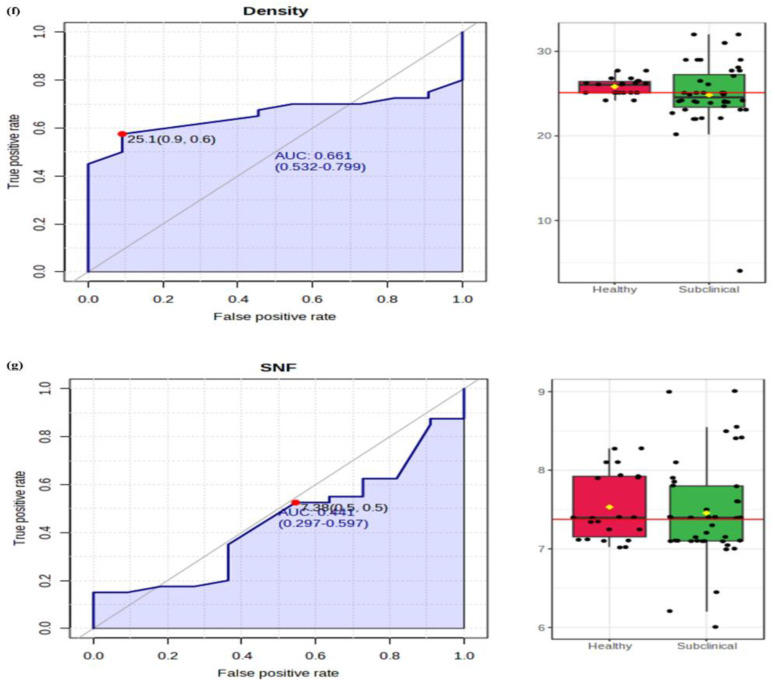
Graphical summary distribution of milk chemistry; (**a**) EC, (**b**) pH, (**c**) protein, (**d**) lactose, (**e**) fat, (**f**) density, (**g**) SNF. The figure on the left shows AUC that represents the area under curve along with sensitivity (true positive rate) and specificity (false positive rate). The bar plots shown on the right indicate the original values (mean ± SD).

In the present study, receiver operating characteristics (ROC) and box plots (representing original values (mean ± SD) in milk of healthy and SCM animals are shown in Figure 4. ROC is an important diagnostic tool that evaluates the sensitivity (true positive rate) and specificity (false positive rate) of diagnostic tests. Moreover, a cut-off point is identified that is considered as best test value as it significantly decreases the number of negative and false positive results. The accuracy of diagnostic test is provided by area under curve (AUC), and a cut-off value of 1.0 signifies perfect accuracy of test. In the present study, the following AUC were calculated: EC (0.93), pH (0.93), protein (0.78), lactose (0.70), fat (0.66), density (0.66) and SNF (0.55). Similarly, the sensitivity and specificity values were determined as: EC (80% & 80%), pH (80% & 90%), protein (70% & 70%), lactose (60% & 90%), fat (40% & 50%), density (60% & 90%) and SNF (50% & 50%). The values of sensitivity and specificity are quite effective in differentiating healthy animals from SCM infected animals. The AUC, sensitivity, specificity and box plots for milk biochemistry are summarised in Figure 4a–g.

### 2.5. Pathogen Identification

In the present study, 90 isolates were obtained from 110 subclinical mastitis-infected milk samples that included *Staphylococcus aureus* (54) and *E. coli* (30), and six samples displayed mixed infections (Table 6).

#### 2.5.1. Isolation of *Staphylococcus aureus*

**Figure 5:**
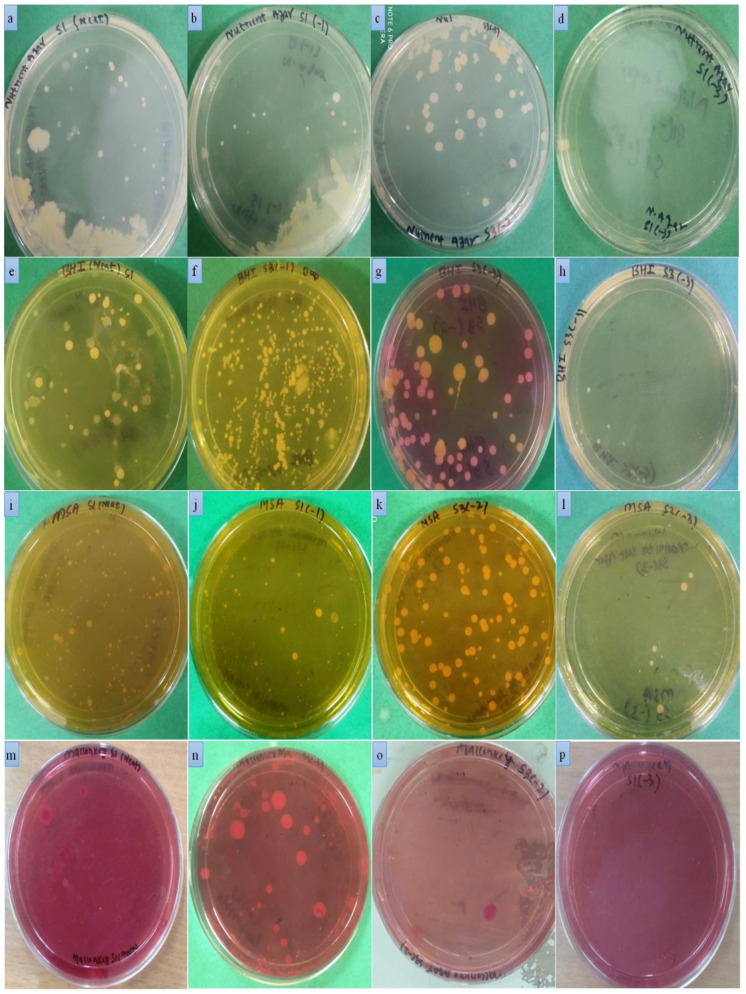

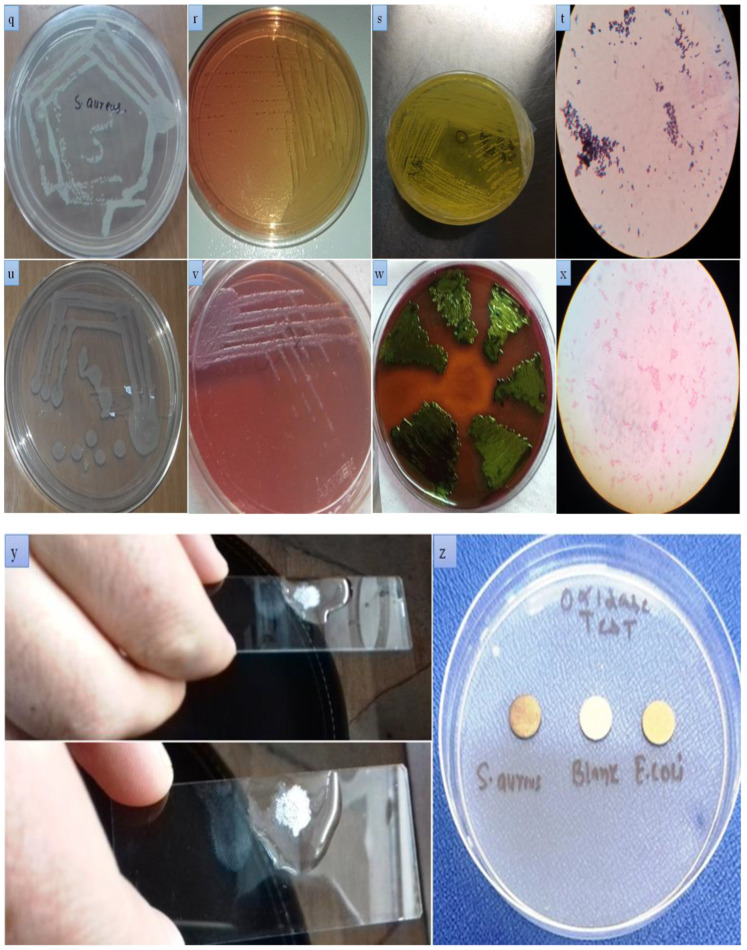
*S. aureus* and *E. coli* on basal and differential media: nutrient agar (**a**–**d**); brain–heart infusion (**e**–**h**); Mannitol salt agar (**i**–**l**); MacConkey agar (**m**–**p**). *Staphylococcus aureus* (nutrient agar (**q**); Mannitol salt agar (**r**–**s**); Gram’s staining (**t**); *Escherichia coli*: nutrient agar (**u**); Mannitol salt agar (**v**); MacConkey agar (**w**); Gram’s staining (**x**); catalase test (**y**) *(S. aureus* and *E. coli*); oxidase test (**z**) (*S. aureus* and *E. coli*).

*S. aureus* was isolated from mastitis-infected milk samples and identified on the basis of growth characteristics, colony morphology, and biochemical methods (Gram’s staining, catalase and oxidase testing). Isolates of *S. aureus* developed golden yellow or white colonies on nutrient agar. Yellow colored colonies were obtained on brain heart infusion agar (BHI). Similarly, on mannitol salt agar (MSA), the color of the media changed from red to yellow because of fermentation. The formation of yellow colonies on MSA with yellow zones is a characteristic growth feature of *S. aureus.* The isolates appeared as Gram positive cocci on Gram’s staining and were arranged in typical grape-like bunches. The isolates tested positive for catalase and negative for oxidase. The growth and biochemical characteristics of *S. aureus* isolates is shown in Figure 5.

#### 2.5.2. Isolation of *E. coli*

*E. coli* isolates were obtained when infected mastitic milk samples were grown on different growth medias. The isolates appeared as transparent, shiny round colonies on nutrient agar. Small pink colored colonies were obtained when isolates were grown on BHI agar. A greenish metallic sheen was developed by *E. coli* isolates when grown on eosin methylene blue agar (EMB agar). Small- to medium-sized pink colored colonies were formed by isolates when grown on MacConkey agar. The formation of metallic colonies on EMB and pink to brick red colonies on MacConkey agar is the characteristic morphological property of *E. coli*. The isolates were further subjected to biochemical identification. Pink colored colonies were formed on Gram’s staining by *E. coli*. A positive catalase test was obtained by observing bubble formation, whereas no changes were observed on the catalase test, which was negative for isolates. The morphological growth characteristics and biochemical features is shown in Figure 5.

### 2.6. In Vitro Antibiotic Sensitivity Test

**Figure 6:**
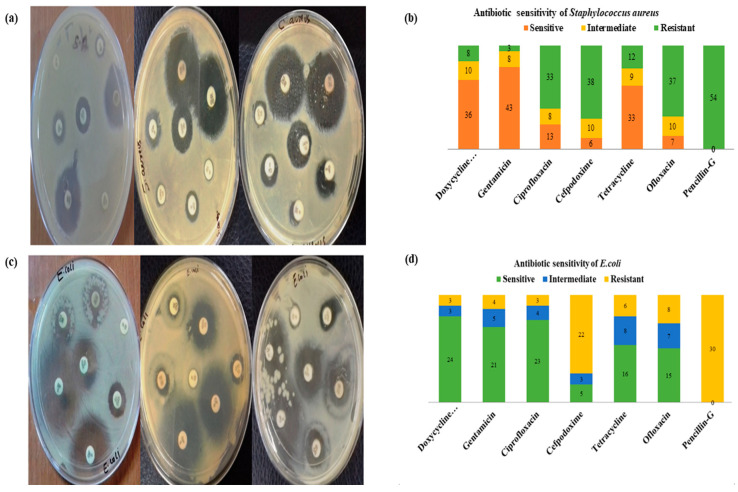
(**a**) Antibiotic sensitivity test of isolated *S. aureus* (Kirby–Bauer method); (**b**) percentage of antibiotic activity for *S. aureus* (DO—Doxycycline hydrochloride, GEN—Gentamycin, CIP—Ciprofloxacin, CPD—Cefpodoxime, TE—Tetracycline, OF—Ofloxacin, PEN—Penicillin; (**c**) antibiotic sensitivity test of the isolated *E. coli* (Kirby–Bauer method); (**d**) percentage of antibiotic activity for *E. coli*. (DO—Doxycycline hydrochloride, GEN—Gentamycin, CIP—Ciprofloxacin, CPD—Cefpodoxime, TE—Tetracycline, OF—Ofloxacin, PEN—Penicillin.

The agar disc diffusion technique was used to evaluate the antibiotic sensitivity of *S. aureus* and *E. coli* isolates. Mueller–Hinton agar (MHA) was used as the media for performing the sensitivity test. *S. aureus* showed sensitivities of 79.6%, 66.6%, and 61.17% to gentamicin, doxycycline hydrochloride, and tetracycline, whereas ciprofloxacin (24.07%), cefpodoxime (11.1%), and ofloxacin (12.9%) were found to be the least sensitive. The highest resistance was shown by isolates to penicillin (100%). The antibiogram results of *S. aureus* is shown in Table 7, whereas Figure 6a,b represents the antibiotic sensitivity and percentage profile of *S. aureus*.

The isolates of *E. coli* showed the highest sensitivity to doxycycline hydrochloride (80%) followed by ciprofloxacin (76.6%) and gentamicin (70%). Cefpodoxime was found to be the least sensitive (16.6%). Among the antimicrobials, isolates of *E. coli* reported the highest resistance to penicillin (100%), followed by cefpodoxime (73.3%).

The antibiogram results of *E. coli* are shown in Table 7, whereas Figure 6c,d represents the graphical sensitivity and percentage profiles of *E. coli* isolates.

In the present study, the major pathogens that were associated with SCM were *S. aureus* and *E. coli* with an overall prevalence of 60% and 33.33%. *S. aureus* was the predominant pathogen of mastitis followed by *E. coli*. The findings of the present study agree with the reports of [27,28]. Different authors have reported the prevalence of *S. aureus* in infected milk as 59.37%, 58%, and 60.87%, respectively [29,30]. *S. aureus* has a wide ecological distribution and is mainly located on the skin and the mammary glands. A high frequency of *S. aureus* infections generally indicates unhygienic practices, poor animal health services, management factors (breed, farm size, no diagnostic facilities, absence of dry cow therapy), and improper attention to mammary gland health, as this pathogen mostly spreads via the milker’s hand.

*E. coli* was the second most common etiological agent of SCM in this study. Similar findings have been reported in previous studies by Jyothi et al. [28] and Demissie et al. [31], who reported 24.4% and 25.7% prevalence, respectively. Studies have reported a higher incidence of *E. coli* with a prevalence of 43.13%, 45.89%, and 41%, respectively [32,33,34]. The higher prevalence of *E. coli* infections indicates poor hygienic conditions, as the pathogen, being environmental, resides in the cow’s surroundings and enters into the udder via the teat canal.

The in vitro antibiotic susceptibility testing of isolates (*S. aureus* and *E. coli*) was performed, which revealed the most effective drug for SCM infections. Isolates of *S. aureus* showed more sensitivity to gentamicin (79.6%) followed by doxycycline hydrochloride (66.6%) and tetracycline (61.1%). Isolates were highly resistant to penicillin (100%), cefpodoxime (70%), ofloxacin (68.1%), and ciprofloxacin (61.1%). The sensitivity findings of this study are in agreement with previous reports [35,36,37]. The reports on isolate resistance to ciprofloxacin, ofloxacin, cefpodoxime, and penicillin are in agreement with previous findings [38,39,40,41,42].

Isolates of *E. coli* showed sensitivities of 80%, 76.6%, 70%, 53.3%, and 50% to doxycycline hydrochloride, ciprofloxacin, gentamicin, tetracycline, and ofloxacin, respectively. The highest resistance was shown by isolates of *E. coli* to penicillin (100%) and cefpodoxime (73.3%). Similar sensitivity findings of *E. coli* to doxycycline hydrochloride, ciprofloxacin, gentamicin, tetracycline, and ofloxacin are in agreement with previous reports [35,36,37]. In the present study, the highest resistance was shown by *E. coli* isolates to penicillin (100%), followed by cefpodoxime (73.3%). These findings are in accordance with previous reports [43,44].

In dairy cattle, continuous antimicrobial use without performing a proper antibiogram of etiological agents is a major cause for treatment failure, thereby leading to drug resistance. Therefore, the antibiogram profiles of isolates may act as vital tools for determining hygienic conditions and health concerns that human beings may face in infections caused by drug-resistant microbes.

## 3. Materials and Methods

### 3.1. Study Area

The present study was carried with a large population of Holstein Friesian crossbred dairy animals were being reared in the study area. The selected animals comprised different age groups (3–10 years old), lactation stages (1–8 months) and had various parity numbers (1–6).

### 3.2. Study Design

In the present study, 135 dairy animals were randomly selected during the period of June 2017 to January 2019. The dairy animals were screened for SCM by cow-side diagnostic tests. The detailed sampling structure is shown in Table 8.

### 3.3. Screening Tests

In the present study, subclinical mastitis animals were screened from the healthy animals through various diagnostic tests, which are mentioned below.

#### 3.3.1. California Mastitis Test (CMT)

This test is a simple available diagnostic test that gives an accurate measure of somatic cells present in milk. CMT is performed by taking milk from all four quarters in a paddle and mixed with the equimolar volume of the reagent (3% sodium dodecyl sulphate). The contents of the paddle are swirled and mixed for 15–20 s. The formation of gel indicates the presence of mastitis infection, whereas no gel formation indicates no infection. This test is based on the underlying principle that the anionic detergent causes the lysis of cell membrane, thereby releasing cell contents, particularly the DNA. This reagent reacts with the cell DNA, thus forming a gel, and the gel intensity determines the severity of infection.

#### 3.3.2. pH

In the present study, the pH in all the milk samples was determined by a portable digital pH meter (Thermo Scientific, Eutech, Singapore). The dairy animals with a pH of greater than 6.7 in milk were grouped as animals with subclinical mastitis, whereas animals with lower values than this were identified as healthy.

#### 3.3.3. Electrical Conductivity (EC)

In milk samples, EC was measured by a portable digital EC meter (Thermo Scientific, Eutech Singapore). The dairy cows with a milk EC higher than 4.44 mS/cm were classed as subclinical mastitis-infected and those with lower values than this were identified as healthy animal.

#### 3.3.4. Somatic Cell Count (SCC)

In SCM, the measurements of SCC in milk is considered as a gold standard test for diagnosis. A portable DeLaval cell counter (DCC; DeLaval International AB, Tumba, Sweden) was used to determine SCC in milk samples. A cut-off point of >200,000 somatic cells per/mL was used for distinguishing SCM-infected cows from healthy animals.

### 3.4. Sample Collection

Milk samples from the selected dairy animals were collected as per the recommendations of [45]. Before the collection of composite milk samples, the udders of animals were properly cleaned with 70% ethanol. About 25 mL of milk was withdrawn aseptically and collected in sterile falcon tubes with few initial milk squirts were discarded. The tubes were labelled accordingly and the relevant information about each animal was entered in a form.

### 3.5. Gas Chromatography–Mass Spectrometry (GC-MS) Analysis

The GC-MS analysis of milk samples was performed at Centre for Ocean Research at the Sathyabama Institute of Science and Technology, Chennai, Tamil Nadu, India. The milk samples were analyzed using a Shimadzu QP2010 Ultra GC-MS. The GC specifications were as follows: column oven temperature, 80 °C; injector temperature, 250 °C; injection mode, split; flow control mode, linear velocity; column flow, 1 mL/min; purge flow, 3 mL/min; split ratio, 5. The temperature was programmed from 80 °C with an increase of 3 °C/min to 200 °C with further increment of 10 °C to 200 °C and then held for 5 min. The MS specifications were as follows: ion source temperature, 240 °C; interface temperature, 250 °C; solvent cut time, 2 min; scan range, 45–900 *m*/*z*; event time, 0.30 s; start time, 4 min; end time, 23 min.

### 3.6. Metabolic Pathway Analysis

The metabolites identified in milk of Holstein Friesian dairy cows were subjected to Metaboanalyst 5.0 software for pathway analysis (http://www.metaboanalyst.ca/; last accessed on 16 April 2022). The pathway analysis (pathway enrichment and topology analysis) of metabolites identified in the milk of healthy and subclinical mastitis dairy cows were entered and analyzed by MetaboAnalyst 5.0 software; last accessed on 16 April 2022). The metabolic pathway analysis for the *Bos taurus* library was selected. The algorithms used for over-representation and pathway topology analysis were the hypergeometric test and out-degree topology. The *p* values for the pathways were calculated and were considered significant at *p* < 0.05.

### 3.7. Metabolite Identification

Milk metabolites were identified based on accuracy, and mass spectrometric fragmentation patterns using METLIN (Metabolite and Tandem MS database), PubChem, KEGG (Kyoto Encyclopedia of Genes and Genomes), BMDB (Bovine Metabolome Database), and HMDB (Human Metabolome Database) [46].

### 3.8. Analysis of Chemical Properties of Milk

The estimation of fat, protein, lactose, solids not fat, specific density, electrical conductivity (EC), and pH was performed in milk samples using an automatic milk analyzer (Speedy Lab, Model 4828, Astori^®^ tecnica, Italy). EC and pH in milk samples were measured using a portable digital meter.

### 3.9. Isolation and Identification of Causative Agents

In composite milk samples, the bacteria were isolated according to the guidelines provided by [45]. In a sterile falcon tube, 9 mL of nutrient broth was placed, to which 1ml of raw milk sample was added. The tubes were then incubated for 24 h at 37 °C with vigorous agitation. Following overnight incubation, one or two loopfuls of broth culture were streaked in different growth media (Nutrient agar, Brain heart infusion (BHI), Mannitol salt agar (MSA), Eosin methylene blue (EMB), and MacConkey agar). The tubes were incubated at 37 °C for 24–48 h and after incubation, the well-separated bacterial colonies were streaked and subcultured again in the same growth media to obtain pure colonies. Based on colony morphology, culture characteristics, and staining methods, the isolated bacteria were isolated were identified.

### 3.10. Antibiogram

Antimicrobial susceptibility testing (AST) was used to identify the most effective antibiotics for mastitis treatment. The disk diffusion method was used to perform AST, which was carried out as per the guidelines of Clinical and Laboratory Standard Institute (CLSI, 2008). The zone of inhibition was measured, and the interpretation was performed as per the instructions of manufacture protocol. Against the antimicrobial agents, organisms were labelled as either ‘sensitive, ‘intermediate’, and ‘resistant’.

### 3.11. Preparation of Inoculums

Inoculums were prepared by using a sterile loop to pick at least 4–5 well types of isolated colonies and then dipping them into nutrient broth for incubation at 37 °C for 24 h.

### 3.12. Antimicrobial Susceptibility Test (AST)

The agar disc diffusion technique was used to perform antibiotic sensitivity patterning of the major isolated pathogens. Mueller–Hinton Agar (MHA) was used as a plating media for disc diffusion test. After pouring the media, overnight bacterial cultures were spread evenly on plate surfaces of MHA and left for 40 min at room temperature to solidify. Antibiotic discs purchased from Hi-media were impregnated on plate surfaces at equal and separated distances and incubated for 24–36 h at 37 °C. Following incubation, the zone of inhibition for each antibiotic was measured and compared with reference standard of the manufacturer. Based on the zone of inhibition formation, the isolates were grouped as sensitive (S), intermediate (I), and resistant (R). In this study, commercially available antibiotics that were used are enlisted in Table 9.

### 3.13. Statistical Analysis

MetaboAnalyst an online tool was used for multivariate statistical analysis (principal component analysis (PCA), sparse partial least discriminant analysis (sPLS-DA), and orthogonal partial least square discriminant analysis (oPLS-DA). In these procedures, no normalization or filtering of data was carried out and a confidence interval of 95% was used. MetaboAnalyst 5.0 was used to generate heat maps with unit scale variance and normalization of data, and comparisons were made by methods based on Euclidean distance. A variable importance in the projection (VIP) was used to rank metabolites and the scores discriminated healthy from SCM animals. The MetaboAnalyst tool was also used for univariate data analysis (fold change analysis and *t*-test).

## 4. Conclusions

In conclusion, findings from this study identified important metabolites of fatty acid metabolism and alterations in the milk chemistry of SCM cows. In understanding SCM pathobiology, the identified metabolites and pathways could prove to be diagnostic and predictive disease markers. Electrical conductivity and pH in milk could be quite useful in distinguishing healthy cows from SCM cows. In SCM, the milk profiles of dairy cows are affected, with most parameters decreasing. Alterations in milk composition during and after the disease diagnosis are important parameters that determine milk quality and its use for human consumption. The antibiogram profile revealed *S. aureus* and *E. coli* as the main pathogens causing SCM in dairy cows in the study area. Antibiotic sensitivity profiles revealed that *S. aureus* was more sensitive to gentamicin, whereas doxycycline hydrochloride was the most affective antibiotic against *E. coli* isolates. Both isolates were highly resistant to the penicillin drugs.

## Figures and Tables

**Table 1:** Metabolites found in milk samples by GC-MS analysis.

Retention Time	Name	Molecular Formula	Molecular Weight (g/mol)	Area (%)
19.670	Tetradecanoic acid	C_14_H_28_O_2_	228.37	14.14
22.111	*n*-Hexadecanoic acid	C_16_H_32_O_2_	256.42	56.06
7.082	Hexanoic acid	C_6_H_12_O_2_	116.16	3.13
13.281	*n*-Decanoic acid	C_10_H_20_O_2_	172.26	4.38
16.839	Dodecanoic acid	C_12_H_24_O_2_	200.32	2.71
20.898	Tricosanoic acid	C_23_H_46_O_2_	354.6	2.35
21.866	*cis*-7-Hexadecenoic acid	C_16_H_30_O_2_	254.41	20.47
7.070	Caprylic acid	C_8_H_16_O_2_	144.21	22.14
21.690	2-Hexadecenoic acid, methyl ester, (*E*)-	C_17_H_32_O_2_	268.4	12.58
19.249	Methyl tetradecanoate	C_15_H_30_O_2_	242.4	15.90
23.321	1,2-Benzenedicarboxylic acid, bis (2-ethylhexyl) ester	C_24_H_38_O_4_	390.6	52.78
16.282	Dodecanoic acid, methyl ester (Methyl laurate or Methyl dodecanoate)	C_13_H_26_O_2_	214.34	8.11
21.690	Hexadecanoic acid, methyl ester or Methyl palmitate or Methyl hexadecanoate	C_17_H_34_O_2_	270.5	54.38
17.311	Methyl stearate	C_19_H_38_O_2_	298.5	2.34
22.050	Succinic acid, 2-ethylhexyl geranyl ester	C_22_H_38_O_4_	366.5	3.10
22.005	Hexadecyl nonanoate or Cetyl nonanoate	C_25_H_50_O_2_	382.7	11.53
23.177	Fumaric acid, dodecyl hex-4-yn-3-yl ester	C_22_H_36_O_4_	364.5	27.83

**Table 2:** Results of compound name mapping.

Query	Match	HMDB	PubChem	KEGG	SMILES	Comment
Tetradecanoic acid	Myristic acid	0000806	11005	C06424	CCCCCCCCCCCCCC(=O)O	1
*n*-Hexadecanoic acid	Palmitic acid	0000220	985	C00249	CCCCCCCCCCCCCCCC(=O)O	1
Hexanoic acid	Caproic acid	0000535	8892	C01585	CCCCCC(=O)O	1
*n*-Decanoic acid	Capric acid	0000511	2969	C01571	CCCCCCCCCC(=O)O	1
Dodecanoic acid	Dodecanoic acid	0000638	3893	C02679	CCCCCCCCCCCC(=O)O	1
Tricosanoic acid	Tricosanoic acid	0001160	17085	NA	CCCCCCCCCCCCCCCCCCCCCCC(=O)O	1
*cis*-7-Hexadecenoic acid	Hypogeic acid	0002186	5318393	NA	CCCCCCCC/C=C\CCCCCC(=O)O	1
Caprylic acid	Octanoic acid	0000482	379	C06423	CCCCCCCC(=O)O	1
Methyl hexadecenoate	2-Hexadecenoic acid, methyl ester, (E)	NA	NA	NA	NA	0
Methyl tetradecanoate	Methyl tetradecanoate	0030469	31284	NA	CCCCCCCCCCCCCC(=O)OC	1
Methyl dodecanoate	Methyl dodecanoate	0031018	8139	NA	CCCCCCCCCCCC(=O)OC	1
Methyl hexadecanoic acid	Methyl hexadecanoic acid	0061859	8181	C16995	CCCCCCCCCCCCCCCC(=O)OC	1
Methyl stearate	Methyl stearate	0034154	8201	NA	CCCCCCCCCCCCCCCCCC(=O)OC	1
Succinic acid, 2-ethylhexyl geranyl ester	NA	NA	NA	NA	NA	0
4,8,12,16-Octadecatetraen-1-ol, 4,9,13,17-tetramethyl	NA	NA	NA	NA	NA	0
Fumaric acid, dodecyl hex-4-yn-3-yl ester	NA	NA	NA	NA	NA	0

Note: 0 indicates no match, 1 indicates exact match, and 2 indicates approximate match.

**Table 3:** Significant metabolic pathways (*p* < 0.05) after single pathway analysis using *Bos taurus* as the library.

	Total	Expected	Hits	Raw *p*	−log(*p*)	Holm Adjust	FDR	Impact
Fatty acid biosynthesis	47	0.21759	5	4.70 × 10^−7^	6.3281	3.95 × 10^−5^	3.95 × 10^−5^	0.0145
Biosynthesis of unsaturated fatty acids	36	0.16667	1	0.15551	0.80824	1	1	0
Fatty acid elongation	39	0.18056	1	0.16748	0.77605	1	1	0
Fatty acid degradation	39	0.18056	1	0.16748	0.77605	1	1	0.0204

**Table 4:** Over-representation analysis of compounds in different organs.

	Total	Expected	Hits	Raw *p*	Holm *p*	FDR
Epidermis	216	2.6	5	0.0958	1	1
Thyroid Gland	12	0.145	1	0.136	1	1
Prostate	267	3.22	3	0.665	1	1
Bladder	87	1.05	1	0.668	1	1
Platelet	108	1.3	1	0.75	1	1
Skeletal Muscle	123	1.48	1	0.796	1	1
Kidney	164	1.98	1	0.886	1	1
Spleen	170	2.05	1	0.896	1	1
Fibroblasts	183	2.2	1	0.914	1	1
Liver	234	2.82	1	0.961	1	1

**Table 5:** Univariate analysis results for each variable/metabolite.

Name	Mean (SD) of 0 (Healthy)	Mean (SD) of 1 (Subclinical)	*p*-Value	Fold Change	0/1	*p* Value Origin
Fat	3.954 (0.828)	3.353 (0.858)	0.0411 (W)	1.18	Up	0.0411
Protein	3.517 (0.466)	3.057 (0.346)	0.0002 (W)	1.15	Up	0.0002
Lactose	4.226 (0.183)	3.909 (0.540)	0.0082 (W)	1.08	Up	0.0082
SNF	7.532 (0.430)	7.455 (0.653)	0.4549 (W)	1.01	Up	0.45488
Specific Density	25.835 (0.983)	24.852 (4.394)	0.0424 (W)	1.04	Up	0.04235
EC	3.382 (0.213)	3.913 (0.361)	<0.0001 (W)	−1.16	Down	0
pH	6.282 (0.404)	6.848 (0.165)	<0.0001 (W)	−1.09	Down	0

**Table 6:** Incidence of pathogens isolated from the milk of subclinical mastitis animals.

Bacteria	Total	No. of Pathogens Isolated	Prevalence %
*Staphylococcus aureus*	90	54	60
*E. coli*		30	33.33
Mixed infections		6	6.67

**Table 7:** In vitro antibiogram of *S. aureus* isolates (*n* = 54) and *E. coli* (*n* = 30) in comparison with various antibiotics.

Isolate	Antibiotics	Sensitive	Intermediate	Resistant
*S. aureus*	Doxycycline hydrochloride	36 (66.6)	10 (18.51)	8 (14.81)
	Gentamicin	43 (79.6)	8 (14.81)	3 (5.55)
	Ciprofloxacin	13 (24.07)	8 (14.81)	33 (61.11)
	Cefpodoxime	6 (11.11)	10 (18.51)	38 (70.37)
	Tetracycline	33 (61.11)	9 (16.66)	12 (22.22)
	Ofloxacin	7 (12.96)	10 (18.51)	37 (68.51)
	Penicillin-G	-	-	54 (100)
*E. coli*	Doxycycline hydrochloride	24 (80%)	3 (10%)	3 (10%)
	Gentamicin	21 (70%)	5 (16.66%)	4 (13.33%)
	Ciprofloxacin	23 (76.6%)	4 (13.33%)	3 (10%)
	Cefpodoxime	5 (16.66%)	3 (10%)	22 (73.33%)
	Tetracycline	16 (53.33%)	8 (26.66%)	6 (20%)
	Ofloxacin	15 (50%)	7(23.33%)	8 (26.66%)
	Penicillin-G	-	-	30 (100%)

**Table 8:** Sample collection from Ganderbal district in the state of Jammu and Kashmir.

Group of Animals	Ganderbal
Healthy	25
Sub-clinical mastitis	110
Total	135

**Table 9:** Antibiotics with their concentration.

S. No	Antibiotic	Concentration
1	Doxycycline hydrochloride (DO)	30 µg
2	Gentamicin (GEN)	10 µg
3	Ciprofloxacin (CIP)	5 µg
4	Cefpodoxime (CPD)	10 µg
5	Tetracycline (TE)	30 µg
6	Ofloxacin (OF)	5 µg
7	Penicillin-G (P)	10 units

## Data Availability

All the data generated during this study are published in this article.
